# Residential Radon and Brain Tumour Incidence in a Danish Cohort

**DOI:** 10.1371/journal.pone.0074435

**Published:** 2013-09-16

**Authors:** Elvira V. Bräuner, Zorana J. Andersen, Claus E. Andersen, Camilla Pedersen, Peter Gravesen, Kaare Ulbak, Ole Hertel, Steffen Loft, Ole Raaschou-Nielsen

**Affiliations:** 1 Diet, Genes and Environment, Danish Cancer Society Research Centre, Copenhagen, Denmark; 2 Construction and Health, Danish Building Research Institute, Aalborg University, Aalborg, Denmark; 3 Department of Public Health, Faculty of Health Sciences, Copenhagen University, Copenhagen, Denmark; 4 Center for Nuclear Technologies, Technical University of Denmark, Roskilde, Denmark; 5 Geological Survey of Denmark and Greenland, Copenhagen, Denmark; 6 National Institute of Radiation Protection, Herlev, Denmark; 7 Department of Environmental Science, Aarhus University, Aarhus, Denmark; Indiana University, United States of America

## Abstract

**Background:**

Increased brain tumour incidence over recent decades may reflect improved diagnostic methods and clinical practice, but remain unexplained. Although estimated doses are low a relationship between radon and brain tumours may exist.

**Objective:**

To investigate the long-term effect of exposure to residential radon on the risk of primary brain tumour in a prospective Danish cohort.

**Methods:**

During 1993–1997 we recruited 57,053 persons. We followed each cohort member for cancer occurrence from enrolment until 31 December 2009, identifying 121 primary brain tumour cases. We traced residential addresses from 1 January 1971 until 31 December 2009 and calculated radon concentrations at each address using information from central databases regarding geology and house construction. Cox proportional hazards models were used to estimate incidence rate-ratios (IRR) and 95% confidence intervals (CI) for the risk of primary brain tumours associated with residential radon exposure with adjustment for age, sex, occupation, fruit and vegetable consumption and traffic-related air pollution. Effect modification by air pollution was assessed.

**Results:**

Median estimated radon was 40.5 Bq/m^3^. The adjusted IRR for primary brain tumour associated with each 100 Bq/m^3^ increment in average residential radon levels was 1.96 (95% CI: 1.07; 3.58) and this was exposure-dependently higher over the four radon exposure quartiles. This association was not modified by air pollution.

**Conclusions:**

We found significant associations and exposure-response patterns between long-term residential radon exposure radon in a general population and risk of primary brain tumours, adding new knowledge to this field. This finding could be chance and needs to be challenged in future studies.

## Introduction

Brain tumours are rare; however incidence rates in Nordic countries have increased during the past few decades in both men and women [1], [2]. The increased incidence rates may partially be explained by improved diagnostic methods and clinical practice [3], but remain largely unknown. Epidemiological studies have investigated many potential risk factors for brain tumour over the past several decades, but the only established cause is ionizing radiation given in therapeutic [4]–[7] and diagnostic doses [8] and data from atomic bomb survivors support this [9], [10]. Residential radon is responsible for the majority of exposure to ionizing radiation in the general population, and although doses are several orders of magnitude lower than doses from therapeutic treatments, the same mechanisms of damage to the brain are expected.

Exposure to radon and alpha emitters polonium-218 and polonium-214 [11] has been classified as a human carcinogen [12]. Radon-222 gas arises from the radioactive decay of radium-226, present throughout the earth’s crust and in many building materials. Radon-222 has a 3.8-day half-life, and builds up indoors where most exposure to the general population occurs. The airways and lungs are the primary target organs, but dose calculations predict that inhaled radon gas and radon progeny can pass the blood-brain-barrier [13] and although estimated brain doses are low, a relationship between residential radon and brain tumours may exist. Yet, little attention has been given to this possibility; three studies of miners exposed to elevated occupational levels of radon have investigated mortality, but report conflicting results, with one showing increased brain cancer mortality related to radon exposure [14] and the others showing reduced mortality [15], [16], all statistically insignificant. A more recent study of uranium miners including 14 deaths due to brain tumours found an excess risk associated with radon exposure although with no dose-response relationship and expressing caution of possible diagnostic misclassification [17]. No study to date has investigated the association between incidence of brain tumour and exposure to residential radon in the general population.

A recent cohort study suggested that the risk of brain tumour could be associated with air pollution at the residential address [18]. Traffic related particulate matter (PM) in ambient air penetrates homes and contributes significantly to indoor PM [19]. Presence of indoor PM may modify the association between residential radon and brain tumours. Unattached radon progeny with an aerodynamic diameter around 1 nm have a high extrathoracic deposition, including the nasal cavity [13]; whilst radon progeny easily attach to PM in the air [20], [21]. The attachment of radon progeny to aerosols in the air reduces the fraction of the so-called unattached radon decay products and increases the airborne concentration of attached radon decay products due to a significant reduction in the plate out on indoor surfaces [22]. Attachment furthermore significantly influences the deposition pattern in the lungs due to the altered size distribution of the radon decay products [23] and experimental evidence supports the theory that ultrafine PM can reach the brain both via the systemic circulation through the blood-brain barrier and via the olfactory neuronal pathway [24]–[26]. Also, radon uptake from airways might be enhanced by exposure to traffic airway irritants including nitrogen dioxide (NO_2_) which is also a marker of PM from traffic. We have previously reported a non-significant pattern of stronger associations between radon and leukaemia among children living at streets with high traffic density [27]; indicating that residential radon and indoor PM might operate together and influence risk.

The “Diet, Cancer and Health” cohort is a large prospective study with detailed information on potential confounders collected at baseline with little potential for recall bias and the radon regression model has been successfully validated [28] and applied in three previous epidemiological studies [27], [29], [30]. Our purpose was to investigate the association between predicted levels of residential radon at the 168,624 residencies of the cohort members, over a period of 39 years and the risk for primary brain tumour in Denmark and to examine the potential modifying effects of air pollution.

## Methods

### Design

Between December 1993 and May 1997, 57,053 persons aged 50 to 64 years were enrolled in the prospective study “Diet, Cancer and Health”. The participants had to be born in Denmark, live in Copenhagen or Aarhus, and cancer free at the time of inclusion [31]. The baseline examination included a self-administered questionnaire on diet including fruit and vegetable consumption, occupational history, including occupation in the chemical industry as well as other items related to health, lifestyle and socio-economic status.

Since establishment of the Danish Civil Registration System (CRS) [32] in 1968, all citizens of Denmark have been given a unique personal identification number, which allows accurate linkage between registers. The CRS is continuously updated regarding many person variables including vital status, place of residence and information on emigration. We traced the date of death, emigration or disappearance of cohort members in the CRS by use of the personal identification number. We retrieved the unique past and present addresses of each participant from 1 January 1971 until 30 December 2009 from the CRS, thus including 39 years of address history dating back to when these cohort members were in their 20 to 40′s. Addresses were identified according to municipality, town, postal code, street, building number, and floor.

We followed each cohort member for occurrence of any cancer from enrolment until 30 December 2009 in the Danish Cancer Registry, which provides accurate and virtually complete nationwide ascertainment of cancers since 1943, including benign tumours [33], by use of the unique personal identification number. Cancers were classified according to ICD-10 (international classification of diseases, 10^th^ revision).

The Scientific Ethics Committee for Copenhagen and Frederiksberg and The Danish Data Protection Agency approved the study, and written informed consent was obtained from all participants prior to enrolment.

### Exposure assessment

Residential radon concentrations at each address of all participants were predicted with a validated regression model [28]. The model uses nine explanatory variables, including geographic location, geology (soil types) and dwelling characteristics including type of house, floor level, total number of floors, fraction of inhabitable space in top floor, basement and building materials. All explanatory variables are available from central Danish databases.

Geographical coordinates were identified by the Danish Geodata Agency by linking the identified unique addresses for all cohort members to the cadastral register, which is a database of all official addresses and their geoordinates in Denmark. The overall goal of the Danish Geodata Agency is to supply and insure reliable and accurate maps and geographical coordinates on all parts of the Realm. Geographical coordinates were obtained for 94% of all the addresses the cohort members had lived in.

The Geological Survey of Denmark and Greenland identified the local soil from digital soil maps using geographical coordinates for each address. House construction data were obtained from the Building and Housing Register [34]. Model predictions were corrected for seasonal variation. The model predicts low level residential radon with great certainty and detects differences in groups well and a comparison with independent radon test data shows that the model makes sound predictions (R^2^ = 0.5) and that errors of radon predictions are only weakly correlated with the estimates themselves [28].

Two radon exposures were calculated for each cohort member from 1 January 1971 onwards. The first was a time-weighted average exposure and the second was a cumulated radon exposure. Both were calculated with and without a 10-year latency period that is relevant for brain tumours. These concentrations were entered into their respective statistical cancer risk models as time-dependent variables; thus recalculating exposure for non-censored persons at the time of each censor.

Information on traffic has previously been collected for the entire study population and traffic-related air pollution has been significantly linked to brain tumours [18]. We estimated the concentration of nitrogen oxides (NO_x_) which correlates strongly with concentrations of ultrafine particles in Danish streets through a wide range of particle sizes (R^2^>0.83) [35] and also includes the airway irritant NO_2_. The average concentrations of NO_x_ at the front door of each dwelling during the period that the participants occupied the address were estimated by use of the Danish air pollution dispersion modeling system, with high temporal and spatial resolution (R^2^>0.75) [36] and including the state-of-the-art Operational Street Pollution Model, currently used in over 17 countries worldwide [37]. We calculated the time-weighted average NO_x_ concentrations at each cohort member’s residential addresses from 1 January 1971 onwards.

### Statistical methods

The end-point for the risk analyses was primary brain tumours, including benign tumours (ICD-10 C71, D330–D332 and D430–D432). Incidence rate-ratios (IRRs) were estimated by a Cox proportional hazards model with age as the underlying time scale ensuring risk estimates were based on individuals at exactly the same age [38]. We calculated two-sided 95% confidence intervals (CIs) on the basis of the Wald test statistic for regression parameters in Cox regression models with the PHREG procedure in SAS (version 9.2; SAS Institute, Cary, NC). Analyses were corrected for delayed entry at the time of enrolment, so that persons were considered under risk from time of enrolment into the cohort. People diagnosed with cancer before enrolment into cohort (except non-melanoma skin cancer) were excluded from the analyses. Censoring occurred at the time of death, emigration or disappearance, cancer diagnosis, or 30 December 2009 (end of follow-up), whichever came first.

Data were analyzed with and without adjustment for a-priori determined variables. The crude model was adjusted for age (underlying time scale) and sex. The second model was further adjusted for individual variables with confounding potential, based on previous literature including: consumption of fruit and vegetables (linear, g/day) [39]–[43], a dichotomous variable indicating employment for at least one year in the chemical industry [12], [44], [45] and NO_x_ at residencies since 1971 (linear, µg/m^3^) [18]. Consumption of fruit and vegetables [39]–[43] and occupation in the chemical industry [12], [44], [45] have been linked to brain tumour; whilst smoking, alcohol and body mass index have consistently been reported to have no effect, despite their association with many other cancer types [4], [7]. The third explorative model was further adjusted for socio-economic variables known to be risk factors for many other cancers, including length of school attendance (<8, 8–10 and >10 years), marital status (single, married/de facto relationship, divorced and widowed) and occupational status (employed versus unemployed). Cohort members that had a missing value for any covariate were excluded, thus ensuring the same number of persons in crude and adjusted analyses.

The assumption of linearity for the continuous variables (residential radon, fruit and vegetable consumption and NO_x_ in relation to brain tumour was evaluated graphically using linear splines with boundaries placed at the nine deciles among all participants as well as by a numerical likelihood ratio test statistic to compare the model assuming linearity with the linear spline model. None of these co-variates deviated significantly from linearity.

We formed four intervals for exposure to residential radon using the 25^th^, 50^th^ and 75^th^ percentiles for all participants as the cut-off points and estimated the IRRs for primary brain tumour for the higher exposure ranges compared with the lowest exposure range. IRRs were also estimated as linear trends in residential radon concentrations. The possible effect modification by traffic-related air pollution was evaluated by introducing interaction terms into the adjusted model and using the Wald’s test.

Exposure-response curves with 95% confidence limits were visualized using a restricted cubic spline in R (library Survival and Design, version 2.13.1), adjusting for age, sex, consumption of fruit and vegetables, employment for at least one year in the chemical industry and NO_x_ at residencies since 1971 [46].

## Results

Among the 57,053 cohort members, we excluded 571 due to a cancer diagnosis before enrolment, 2 because of uncertain date of cancer diagnosis, 960 for which address history was not available in the CRS or their baseline address could not be geocoded, 1,603 because of missing data in potential confounders, and 2,243 because radon or NO_x_ exposure was assessed for less than 80% of the time from 1 January 1971 until diagnosis or censoring. The 51,674 included cohort members had lived in a total of 168,684 addresses and were followed up for cancer for an average of 12.6 years (total person years at risk was 652,028). We identified 121 primary brain tumour cases, corresponding to an overall incidence rate of 18.6 per 100,000 person-years.

Table 1 shows the characteristics of the cohort members and the primary brain tumour cases. Sex distribution and marital status were similar among cases and cohort members. The proportion of participants with employment and long school attendance was slightly lower among cases than among the cohort members and cases consumed slightly less fruit and vegetables. The median predicted residential radon concentration was slightly higher for cases (41.8 Bq/m^3^) than the whole cohort (40.5 Bq/m^3^) and median NO_x_ concentrations were similar for cases and the cohort members and the 95 percentile values for both radon and NO_x_ exposure was higher for cases. Table 1 also shows that those living at addresses with high radon tended to: be men, be employed, have longer school attendance, be married or live in de facto relationships and be exposed to lower NO_x_ levels.

**Table 1:** Characteristics of all study participants, cases and those with low and high levels of radon at the residences.

Characteristic	Cohort		Cases		Radone,c <67.6 Bq/m^3^		Radone,c <67.6 Bq/m^3^	
	No. (%)	Median (5–95 percentile)	No. (%)	Median (5–95 percentile)	No. (%)	Median (5–95 percentile)	No. (%)	Median (5–95 percentile)
All participants	51 674 (100)		121* (100)		38 763 (75.0)		12 911 (25.0)	
Age at enrolment		56.1 (50.7–64.2)		57.2 (50.9–64.2)		56.1 (50.7–64.2)		56.5 (50.9–64.2)
Sex								
Male	24 533 (47.5)		56 (46.3)		18 067 (46.6)		6 466 (50.1)	
Female	27 141 (52.5)		65 (53.7)		20 696 (53.4)		6 445 (49.9)	
Occupational status								
Employed	40 314 (78.0)		89 (73.5)		30 010 (77.4)		10 306 (79.8)	
Unemployed	11 360 (22.0)		32 (26.5)		8 753 (22.6)		2 605 (20.2)	
School attendance (years)								
<8	16 924 (33.0)		54 (44.6)		13 130 (33.9)		3 794 (29.4)	
≥8	34 750 (67.0)		67 (55.4)		25 633 (66.1)		9 117 (70.6)	
Marital status								
Single	3 028 (5.9)		10 (8.3)		2 832 (7.3)		196 (1.5)	
Married/living de facto	37 225 (72.0)		87 (71.9)		26 180 (67.5)		11 045 (85.6)	
Divorced	8 581 (16.6)		17 (14.1)		7 510 (19.4)		1 071 (8.3)	
Widowed	2 840 (5.5)		7 (5.8)		2 241 (5.8)		599 (4.6)	
Fruit and vegetable intake (g/day)		312 (96–735)		282 (75–617)		308 (91–744)		322 (115–713)
Enployment in chemical industry^a^								
No	51 446 (99.6)		119 (98.4)		38 587 (99.6)		12 859 (99.6)	
Yes	228 (0.4)		2 (1.6)		176 (0.4)		52 (0.4)	
Radon at the addressb,c (Bq/m^3^)		40.5 (9.1–91.0)		41.8 (8.8–92.2)				
NO_x_ at front door^d^ (µg/m^3^)		21.6 (14.8–67.7)		22.0 (15.2–92.9)		23.1 (15.3–76.6)		17.5 (14.4–31.8)

^a^Ever employed in the chemical industry for at least 1 year.

b Time-weighted average radon.

c For the period 1 January 1971, to censoring date with inclusion of a 10-year latency period relevant for brain tumour.

d Time-weighted average NO_x_, we focused on the concentration of NO_x_ as an indicator for particular matter (PM) from traffic because NO_x_ correlates strongly with ultrafine particles in Danish streets [35].

e Cut-off based on the 75^th^ percentile for time-weighted average radon concentrations.

* Including 11 cases of benign tumours.

**Figure 1:**
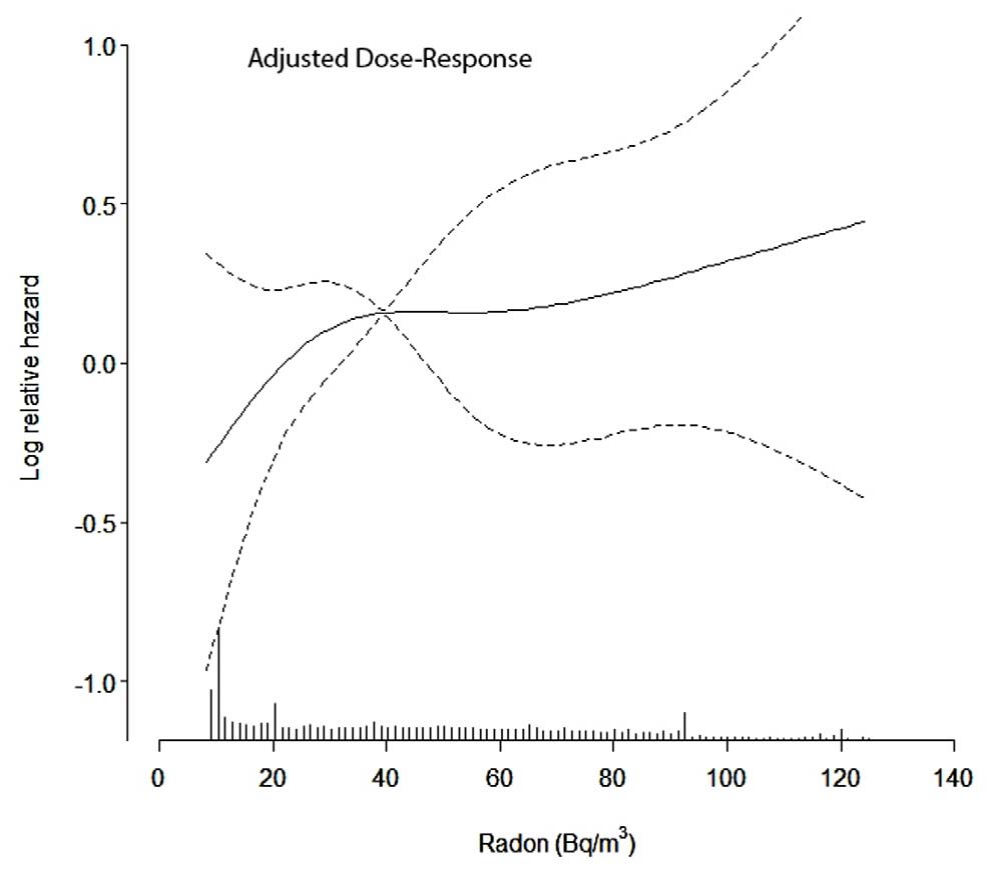
The spline function is adjusted for age, sex, consumption of fruit and vegetables, employment in the chemical industry for at least one year and traffic-related air pollution. The exposure distribution of average residential radon is marked on the x-axis. The spline function can be interpreted as the exposure-response association. The difference between two points on the y-axis on the curve is interpreted as the difference in loge(IRR) for the corresponding difference in exposure, which can be read on the x-axis between the same two points.

Overall the adjusted IRR associated with each 100 Bq/m^3^ increment increase in average radon levels was 1.96 (95% CI: 1.07; 3.58) and the adjusted IRR associated with a 10^3^ Bq/m^3^-years increment increase in cumulated radon was 1.37 (95% CI: 1.03; 1.82). The IRRs for both average and cumulated radon exposure were exposure-dependently higher over the four radon exposure quartiles (Table 2). The unadjusted results showed lower IRR associated with radon levels but the IRRs were also dose-dependently higher over the four-radon quartiles (Table 2). Traffic related air pollution was the most important co-variate for the change in the estimated association between radon and brain tumour risk in model 2 and the crude model. When exploring the effects of co-variates related to socio-economic status in an extended model we found IRRs for the association increased further, primarily due to adjustment for length of schooling (Table 2). These risks estimated were only affected to a small extent by exclusion of the 10-year latency period for radon exposure (results not shown). Figure 1 shows the adjusted exposure-response between average residential radon concentrations and primary brain tumour risk shown in Table 2. There was no evidence that the association between radon and risk of primary brain tumour was modified by traffic-related air pollution, although the point estimate of the IRR was lower among subjects with high levels of NO_x_ at their residence (*p-*value for interaction = 0.15) (Table 3).

**Table 2:** Incidence rate ratios (95% CI) for primary brain tumour risk associated with the residential radon concentrations.

Radon	Cases, n	IRR (95% CI)^b^		
		Crude^c^	Model 2c,d	Model 3c,d.e
Time weighted average (Bq/m^3^)^a^				
<17.2	28	1.00	1.00	1.00
17.2–40.5	29	1.20 (0.69–2.08)	1.44 (0.82–2.54)	1.53 (0.87–2.71)
40.5–67.6	31	1.27 (0.74–2.20)	1.68 (0.94–3.01)	1.86 (1.03–3.38)
>67.6	33	1.38 (0.82–2.36)	1.90 (1.07–3.39)	2.09 (1.16–3.79)
*Linear trend per 100 Bq/m^3^*	*121*	*1.43 (0.81–2.55)*	*1.96 (1.07–3.58)*	*2.15 (1.16–4.01)*
Cumulated exposure (Bq/m^3^-years)^a^				
<377	29	1.00	1.00	1.00
377–895	31	1.14 (0.69–1.97)	1.40 (0.81–2.42)	1.50 (0.86–2.60)
895–1506	30	1.16 (0.67–1.94)	1.49 (0.84–2.63)	1.64 (0.92–2.94)
>1506	31	1.41 (0.83–2.39)	1.92 (1.08–3.40)	2.11 (1.17–3.81)
*Linear trend per 10^3^Bq/m^3^years*	*121*	*1.19 (0.90–1.56)*	*1.37 (1.03–1.82)*	*1.44 (1.07–1.93)*

a From 1 January 1971 until censoring, with inclusion of a 10 year latency period. The cut-off points between exposure groups were the 25^th^, 50^th^ and 75^th^ percentiles for all participants.

b Analyses based on 51,674 cohort members and 121 brain tumours.

c Adjusted for age by using it as the underlying time scale in the Cox model and sex.

d Adjusted for consumption of fruit and vegetables, employment in the chemical industry for at least one year and traffic (time-weighted average NO_x_ exposure between 1971 and the censoring date).

e Adjusted for employment status, schooling and marital status. Due to exclusion of cohort members with missing value in any covariate, the number of persons is identical in the crude and the adjusted analyses.

**Table 3:** Adjusted^a^ incidence rate ratios for primary brain tumour in association with a 100 Bq/m^3^ increase in domestic radon^b^ within strata of NO_x_ at the residential address.

Potential effect modifier	Cases, n	IRR (95% CI)	P^c^
NO_x_ at front door (µg/m^3^)^d^			
<21.6	58	2.53 (1.06–6.04)	0.15
≥21.6	63	0.98 (0.39–2.50)	

a We adjusted the analyses for age (underlying time scale), sex, employment in the chemical industry for at least one year and consumption of fruit and vegetables.

b Radon exposure was entered as a continuous variable in all models as the time-weighted average concentration at residences from 1. January 1971 until censoring.with inclusion of a 10 year latency period.

c Test of the null hypothesis that the linear trends are identical, for Wald test for interaction.

d Time-weighted average concentration for NO_x._

## Discussion

We found significant associations and exposure-response patterns between long-term exposure to residential radon in a general Danish population and primary brain tumour risk.

The strengths of this study include a prospective follow-up where information on potential confounding factors was collected at enrolment without potential for recall bias. Complete follow-up for cancer, vital status as well as address history from 1971 onwards was ensured by use of reliable population-based Danish registries. The use of a recently developed regression model facilitated estimation of residential radon in as many as 168,624 homes, over almost four decades. The model has been applied in three previous epidemiological studies [27], [29], [30] and successfully validated against independent radon measurements [28]. Model-based estimation of radon is inevitably associated with uncertainty [28] and it is clear that measurements in homes would provide a more accurate assessment of radon concentrations. But use of measurements in epidemiological studies may imply disadvantages such as a limited number of measurements due to economy constraints and exposure misclassification when reconstructing past residential radon exposures. The advantage of our model-based estimation of radon levels is the facilitation of a larger study with historical estimates of radon exposure since 1971, at reasonable costs. Limitations of our study include the limited number of cases, which prevented analyses of association for specific neuroepithelial/astrocytic tumours. Also, the possibilities of therapeutic exposures at large doses were not considered here. Finally, the exposure of cohort members before 1971 could not be estimated, as residential histories before that date were unknown. Therefore, we were unable to assess early-life radon exposure which is an important limitation as early life environmental exposures might be most significant for cancer risk.

In the present study we show an exposure-response association between residential radon and a risk of primary brain tumour that was almost doubled per each 100 Bq/m^3^ increment in average long-term residential radon exposure. This adds novel information to this field as no study to date has been conducted on the relationship between brain tumour risk and residential radon exposure of the general population.

Models predicting the dose possibly reaching the brain after alveolar uptake from radon exposure indicate that this is equivalent to less than 0.15 mSv per year from 200 Bq/m^3^ [13]. The risk we found associated with residential radon exposures for up to 39 years cannot be explained by a cumulative dose to the brain from transport through the blood and a simple dose-response extrapolation from high-level exposures. An alternative explanation might be that especially unattached radon progeny with aerodynamic diameter around 1 nm and a high extrathoracic deposition including the nasal cavity could reach the brain via the olfactory neuronal pathway [25], [47] resulting in local intense exposure. If this is the explanation for our finding one could, however, have expended a more clear association between the higher radon exposure of miners and risk for brain tumours than found in the four previous studies of miners, although the different particle size distribution in the mines compared to residences could make the olfactory neuronal pathway less relevant in the mines. However, this is hypothetical and our findings could be a result of chance and should be challenged by more studies.

We hypothesised that the presence of PM modifies the association between residential radon and risk of brain tumour and tested the hypothesis with respect to outdoor traffic-related air pollution at the residence which can penetrate indoors [19] as a marker of indoor PM, but our results did not support this hypothesis. In fact we found a stronger association between radon and brain tumour at low outdoor NOx concentrations, although the effect modification was not significant. Hypothetically, presence of traffic emission particles mainly in the size range from around 20 nm, could reduce the availability of unattached radon progeny, which would reduce upper airway deposition and could thus be of importance if the olfactorial neuronal pathway is of relevance for brain exposure.

## Conclusion

We found significant association and exposure-response patterns between long-term exposure to residential radon in a general Danish population and risk of primary brain tumour. Our findings could be a result of chance and need to be challenged by future studies.
